# *De novo* transcriptome assembly, annotation and comparison of four ecological and evolutionary model salmonid fish species

**DOI:** 10.1186/s12864-017-4379-x

**Published:** 2018-01-08

**Authors:** Madeleine Carruthers, Andrey A. Yurchenko, Julian J. Augley, Colin E. Adams, Pawel Herzyk, Kathryn R. Elmer

**Affiliations:** 1 Institute of Biodiversity, Animal Health & Comparative Medicine, College of Medical, Veterinary & Life Sciences, University of Glasgow, G12 8QQ, Glasgow, UK; 2 Glasgow Polyomics, Wolfson Wohl Cancer Research Centre, University of Glasgow, G61 1QH, Glasgow, UK; 3 Scottish Centre for Ecology and the Natural Environment, University of Glasgow, Rowardennan, G63 0AW UK; 4 Present Address: Fios Genomics Ltd., Nine Edinburgh Bioquarter, 9 Little France Road, Edinburgh, EH16 4UX UK; 5 Institute of Molecular, Cell & Systems Biology, College of Medical, Veterinary & Life Sciences, University of Glasgow, G12 8QQ, Glasgow, UK

**Keywords:** Salmonids, Transcriptome, RNA-seq, Annotation, BLAST, Gene Ontology (GO) analysis, BUSCO, OrthoFinder

## Abstract

**Background:**

Salmonid fishes exhibit high levels of phenotypic and ecological variation and are thus ideal model systems for studying evolutionary processes of adaptive divergence and speciation. Furthermore, salmonids are of major interest in fisheries, aquaculture, and conservation research. Improving understanding of the genetic mechanisms underlying traits in these species would significantly progress research in these fields. Here we generate high quality *de novo* transcriptomes for four salmonid species: Atlantic salmon (*Salmo salar*), brown trout (*Salmo trutta*), Arctic charr (*Salvelinus alpinus*), and European whitefish (*Coregonus lavaretus*). All species except Atlantic salmon have no reference genome publicly available and few if any genomic studies to date.

**Results:**

We used paired-end RNA-seq on Illumina to generate high coverage sequencing of multiple individuals, yielding between 180 and 210 M reads per species. After initial assembly, strict filtering was used to remove duplicated, redundant, and low confidence transcripts. The final assemblies consisted of 36,505 protein-coding transcripts for Atlantic salmon, 35,736 for brown trout, 33,126 for Arctic charr, and 33,697 for European whitefish and are made publicly available. Assembly completeness was assessed using three approaches, all of which supported high quality of the assemblies: 1) ~78% of Actinopterygian single-copy orthologs were successfully captured in our assemblies, 2) orthogroup inference identified high overlap in the protein sequences present across all four species (40% shared across all four and 84% shared by at least two), and 3) comparison with the published Atlantic salmon genome suggests that our assemblies represent well covered (~98%) protein-coding transcriptomes. Thorough comparison of the generated assemblies found that 84-90% of transcripts in each assembly were orthologous with at least one of the other three species. We also identified 34-37% of transcripts in each assembly as paralogs. We further compare completeness and annotation statistics of our new assemblies to available related species.

**Conclusion:**

New, high-confidence protein-coding transcriptomes were generated for four ecologically and economically important species of salmonids. This offers a high quality pipeline for such complex genomes, represents a valuable contribution to the existing genomic resources for these species and provides robust tools for future investigation of gene expression and sequence evolution in these and other salmonid species.

**Electronic supplementary material:**

The online version of this article (10.1186/s12864-017-4379-x) contains supplementary material, which is available to authorized users.

## Background

Salmonid fishes are globally recognised for their economic and ecological value. Several species, particularly from the genera *Salmo*, *Onchorhynchus* and *Salvelinus*, contribute significantly to the economy through aquaculture, wild stock fisheries and recreational fishing, and to the environment via their promotion of ecosystem function and biodiversity [1, 2]. In addition, salmonids exhibit exceedingly high levels of diversity in their life histories, behaviour, morphology and physiology, with patterns of trait variation often replicated within and across species, as well as across different freshwater systems [3–7]. This makes salmonids particularly interesting in the context of fundamental and applied research on intra- and inter-specific diversity in morphology, physiology and ecology.

**Fig. 1:**
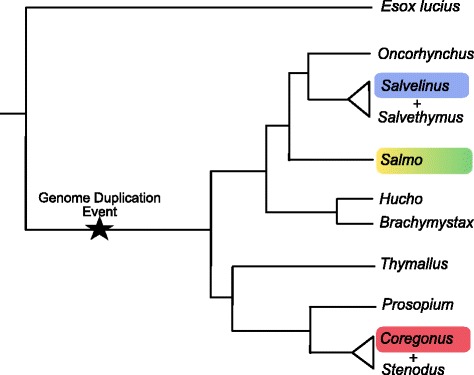
Phylogenetic relationship of salmonids and the closest teleost out-group, *Esox lucius*. Phylogenetic positions and estimated WGD timing follow [11]. The highlighted tree branches represent the phylogenetic positions of species for which assemblies have been generated in the current study, yellow = Atlantic salmon, green = Brown trout, blue = Arctic charr and red = European whitefish

To drive this research forward, we need to understand the genetic basis associated with ecological and evolutionary processes in salmonids. Genetic studies of salmonids are complicated by a whole genome duplication (WGD) event that occurred in their common ancestor approximately 80–100 Mya (Fig. 1; [8–12]). Nevertheless, several important resources have been established through the efforts of consortia such as cGRASP (Consortium for Genomic Research on All Salmonids Program, http://www.sfu.ca/cgrasp/index.html), ICSASG (International Collaboration to Sequence the Atlantic Salmon Genome), and SalmonDB (http://salmondb.cmm.uchile.cl). These include expressed sequence tag (EST) databases, microarray gene expression platforms, and SNP arrays. Consortia efforts have generated extensive EST databases for Atlantic salmon (*Salmo salar*) and rainbow trout (*Oncorhynchus mykiss*) [13–18], as well as on a smaller scale for other salmonid species such as chinook salmon (*Oncorhynchus tshawytscha*), sockeye salmon (*Oncorhynchus nerka*) and lake whitefish (*Coregonus clupeaformis*) [13]. cGRASP have also generated dense microarray (44 K oligo array) and SNP-chip (~130 K) platforms for Atlantic salmon [19–23].

A further major advance in salmonid genomic research, promoted by consortia efforts, is the recent publication of reference genomes for rainbow trout [10] and Atlantic salmon [12]. The release of these reference genomes presents exciting opportunities for tackling key topics in ecological, evolutionary and fisheries research. In addition, they provide a solid platform for generating genomic resources for other salmonid species. The latter point forms the primary objective of the FAASG (Functional Annotation of All Salmonid Genomes), a recent initiative developed by the ICSASG, which aims to generate functionally annotated resources for nine species of salmonids and integrate data generated from within the wider research community [24]. Rapid advances in next-generation sequencing (NGS) technologies and with significant reductions in cost have made high-throughput RNA sequencing of salmonids more accessible. RNA-Seq methods allow genome-wide investigation of the transcriptome, providing an in-depth overview of transcript sequence and expression profiles [25–28]. Improved *de novo* assembly methods enable robust generation of a reference transcriptome and offer an important alternative to genome reference mapping [28–30]. Thus data generated as part of small-scale projects, such as the four species transcriptome resources presented in the current study, provide a valuable contribution to the development of community resources.

Given that there is currently no reference genome available for the vast majority of salmonids, transcriptomes are key to facilitating research on genomic variation and expression. Efforts by independent salmonid research groups have generated *de novo* transcriptome assemblies for Kokanee salmon (*Oncorhynchus nerka*: 11,085 transcripts, [31]), lake whitefish (*Coregonus clupeaformis*: 77,797 transcripts [32]), coho salmon (*Oncorhynchus kisutch*: 43,228 transcripts [33]), and Arctic charr (*Salvelinus alpinus*: 34,690 transcripts [34]). Furthermore, the recent release of the PhyloFish database (http://phylofish.sigenae.org/index.html) represents another major contribution, with the generation of transcriptomic resources for 15 fish species, of which six are salmonids: grayling (*Thymallus thymallus*), lake whitefish (*Coregonus clupeaformis*), European whitefish (*Coregonus lavaretus*), brown trout (*Salmo trutta*), rainbow trout (*Oncorhynchus mykiss*) and brook trout (*Salvelinus fontinalis*), with 66,996 to 78,415 transcripts per species [35]. The high number of transcripts often assembled for salmonids may be due to the additional whole genome duplication event that occurred in salmonids but it is also possible that redundant or fragmented transcripts inflate some assemblies [33, 34]. Assembly filtering methods are key to generating high-quality transcriptomic references because this will in turn optimise subsequent analyses, such as differential gene expression, allele-specific expression, SNP calling, and sequence evolution [36].

In the current study, we generate comprehensive reference transcriptomes for four salmonid species: Atlantic salmon (*Sm. salar*), brown trout (*Sm. trutta*), Arctic charr (*Sv. alpinus*) and European whitefish (*C. lavaretus*) (Fig. 1). We assembled our transcriptomes using well established *de novo* methods to avoid any bias in the initial construction that might have been introduced by a genome-guided approach, given the varying degrees of phylogenetic divergence of our focal species to the two salmonid species for which reference genomes are currently available, rainbow trout [10] and Atlantic salmon [12]. We also conduct a thorough comparison of the *de novo* assemblies generated for these four species, providing valuable insight into the level of sequence similarity and divergence between salmonids of varying phylogenetic proximity. Furthermore, by comparing across four closely related taxa we were able to robustly distinguish the presence of orthologous and paralogous sequences in our transcriptomes. Finally, we apply several methods to assess assembly completeness, including a comparative analysis of the current assemblies against the published reference genome for Atlantic salmon, and other reference transcriptomes available. The new salmonid transcriptomes we present and characterise here make an important contribution to the currently available genomic resources for salmonids, facilitating future analyses and downstream applications of genome annotation, gene expression, and sequence evolution.

## Methods

### Specimens

Parental fish for this study were from different locations, depending on the species. Atlantic salmon were collected from an anadromous river running population on the river Blackwater (northern Scotland), brown trout were third-generation hatchery trout from Houietoun Hatchery (Stirling, Scotland), Arctic charr were wild caught from a generalist freshwater population in Loch Clair (North-west Scotland), and European whitefish were wild caught from the generalist freshwater population at Loch Lomond (central Scotland). Fish collection was undertaken under licence from Marine Scotland and with local permissions, and a licence specifically to collect *Coregonus lavaretus* was granted by Scottish Natural Heritage. Nine full-sib families of Atlantic salmon, 14 full-sib families of brown trout, nine full-sib families of Arctic charr and six full-sib families of European whitefish were generated. Salmonids exhibit highly variable life histories both within and across species (e.g. freshwater resident versus anadromous migratory individuals). All salmonids are born in freshwater and spend their juvenile phase there, irrespective of any subsequent difference in life histories. Here we sampled all individuals within five months of hatching and therefore all specimens included in the present study were free-feeding and still within their freshwater phase [3–7]. Fry of all four species were reared under common hatchery conditions at the Scottish Centre for Ecology and the Natural Environment, Loch Lomond. Tanks used a flow through system using untreated water from Loch Lomond and subject to ambient temperature of the loch, which ranged from 4 to 16 °C over the duration of the study. After 800 degree-days (dd) post-hatch (~ 5 months old) 32 juvenile fish (eight per species) were randomly selected and euthanized by Benzocaine overdose as per a UK Home Office Schedule 1 approved killing method. This work was conducted under Home Office licence number PPL 60/41/91 granted under the UK Animal (Scientific Procedures) Act 1986. All specimens were weighed (0.19 ± 0.06 g) and stored in RNALater (Life Technologies, Carlsbad, CA). To allow permeation of the RNALater preservative into all tissues, several incisions were made along the dorsal side of each specimen before being submerged in the RNALater. All samples were then stored at 4 °C for 24 h and then frozen at −20 °C until RNA isolation was carried out.

### RNA extraction

Total RNA was isolated from liquid nitrogen homogenised samples (multiple replicates per individual, using the entire sample) using PureLink RNA Mini Kits (Life Technologies, Carlsbad, CA), following an adapted protocol from Gunter et al. [37]. Samples were quantified with a Qubit 2.0 fluorometer (Life Technologies, Carlsbad, CA) and quality was assessed with a 2200 Tapestation (Agilent, Santa Clara, CA). All RNA was high quality; A260/280 ratios were between 1.9 and 2.1 and RIN (RNA Integrity Number) values were above 8.5.

### RNA-seq library construction and sequencing

RNA-seq library preparation and sequencing was carried out by Glasgow Polyomics research facility at the University of Glasgow. Briefly, libraries were synthesised for each of the 32 samples using the TruSeq Stranded mRNA Sample Preparation kit (Illumina, San Diego, CA), according to the manufacturer’s instructions. Paired-end sequencing (75 bp from each end) was then performed on the NextSeq 500 system (Illumina, San Diego, CA) at a sequencing depth of 20–25 million reads per library. The raw reads in bcl format were converted to fastq files with Illumina provided bcl2fastq *v*2.15.0 software, and quality was examined using FastQC *v*0.11.2 [38].

### Initial *de novo* assembly

**Fig. 2:**
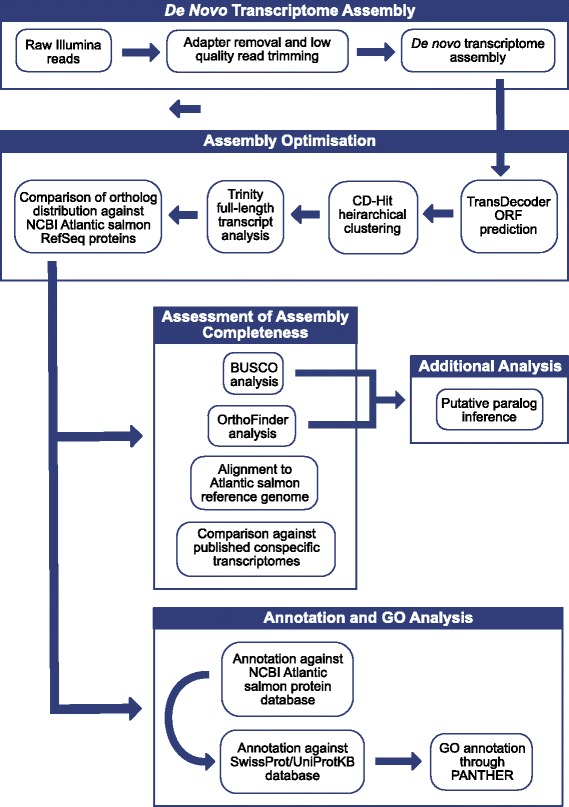
Schematic of the *de novo* transcriptome reconstruction and analysis pipeline used to generate the protein-coding transcriptome assemblies for Atlantic salmon, brown trout, Arctic charr and European whitefish

A schematic representation of the *de novo* transcriptome reconstruction and analysis pipeline is given in Fig. 2. The sequencing reads in fastq format were subjected to pre-processing where adapter sequences were removed with Scythe *v*0.9944 BETA [39] and low quality reads were trimmed with Sickle *v*1.210 [40] (Phred quality score > 30). Given that of the four species studied, a reference genome is currently only available for Atlantic salmon, we assembled the transcriptomes *de novo* to avoid any bias that might have been introduced by a genome-guided approach. Previous studies show that *de novo* generally out-performs genome-guided transcriptome assembly methods for diverged species and for organisms with more complex genomes [41–43]. Given the varying level of phylogenetic divergence of our focal species from Atlantic salmon (Fig. 1, [11]) and the high complexity of the genome (as a result of the Ss4R duplication event [9]), we deemed *de novo* assembly to be more appropriate here. Consequently, the pre-processed reads for each of the four species were subjected to the *de-novo* assembling procedure using Trinity r20140717 [44], with the default parameters. The assembly was performed on Glasgow Polyomics 64-core server with 512Gb RAM.

### Assembly optimisation

Prior to filtering our four *de novo* assemblies to remove redundant and poorly constructed transcripts, we performed an initial quality assessment of the transcript sets. We determined completeness by examining the number of input RNA-seq reads that were represented in our *de novo* assembled transcriptomes, as per the pipeline suggested in the Trinity package (http://github.com/trinityrnaseq/trinityrnaseq/wiki) [45]. Read representation was determined by mapping the cleaned reads back to their corresponding assemblies, for each of the four species individually, with Bowtie2 *v*2.2.6 (--local, --no-unal) [46].

### Removal of redundant transcripts

To obtain sets of non-redundant transcripts we applied the following filtering steps: first, we used TransDecoder v3.0.0 [47] to identify all likely coding regions within our assembled transcripts (for each species individually), and then filtered by selecting the single best open reading frame (ORF) per transcript, as per the TransDecoder pipeline (--single_best_orf). Any transcripts with ORFs less than 200 bp in length were removed before performing further analyses. Second, redundancy was further reduced in the remaining transcript sets by clustering highly similar sequences with CD-Hit *v*4.6.6 [48], using an amino acid sequence identity threshold of 1.00.

### Full-length transcript analysis

To determine how successfully assembled transcripts were reconstructed to full- or near full-length in each of the four assemblies, we calculated coverage against the NCBI Atlantic salmon proteins database (GCF_000233375.1). Atlantic salmon proteins were used as the reference dataset because it is the salmonid species with the most genomic resources available. The non-redundant sets of transcripts were subjected to BLASTP searches (−max_target_seqs 1, −evalue 1e-3) [49] against the Atlantic salmon proteins; we applied a relaxed e-value to avoid discarding good hits for very short sequences. We processed the BLAST hits using the ‘analyze_blastPlus_topHit_coverage.pl’ script from the Trinity package (http://trinityrnaseq.sourceforge.net/) [45] to identify the number of transcripts that aligned to the salmon proteins across varying length thresholds. The results from the Trinity full-length analysis were used to filter the assemblies, excluding all transcripts with less than 30% reference coverage. We used a relatively low coverage threshold to minimise the loss of ‘true’ transcripts from the charr and whitefish datasets, given their increased phylogenetic distance from Atlantic salmon (Fig. 1).

### Assessment of assembly completeness

To provide a comprehensive and quantitative overview of the level of completeness achieved for our assemblies, we applied four approaches to assess overlap with transcriptomic or genomic resources across taxa [50].

First, we quantified completeness by comparing our four assembled transcript sets against a set of highly conserved single-copy orthologs. This was accomplished using the BUSCO (Benchmarking Universal Single-Copy Orthologs) *v*2 pipeline [51] compared to the predefined set of 4584 Actinopterygian single-copy orthologs from the OrthoDB *v*9.1 database [52]. We calculated the number of complete (length is within two standard deviations of the mean length of the given BUSCO), duplicated (complete BUSCOs represented by more than one transcript), fragmented (partially recovered BUSCOs) and missing (not recovered) in each of the four *de novo* assemblies. To further assess the completeness and utility of the resources presented here, we examined how successfully BUSCOs were recovered in our assemblies compared to the NCBI protein dataset for Atlantic salmon (GCF_000233375.1) (48,602 transcripts; based on retaining only the longest isoform per gene), as well as against the PhyloFish brown trout and European whitefish assemblies (75,388 and 74,701 transcripts respectively) [35].

Second, we used the program OrthoFinder *v*1.1.2 [53] to identify orthologous groups of proteins amongst our four assemblies using BLAST all-v-all (self and reciprocal BLASTs simultaneously) algorithm and to further quantify assembly completeness. OrthoFinder represents a novel method of orthogroup detection, by performing reciprocal best-hit BLASTs that are normalised for transcript length, removing transcript length bias in the ortholog detection. OrthoFinder analysis was conducted for all pair-wise comparisons, for all four species assemblies, as well as against the Atlantic salmon RefSeq proteins (GCA_000233375.4) in order to identify putative orthologs within the current datasets and to provide a reference source of identified orthologs and their distribution relative to the existing high-quality protein set for Atlantic salmon. We used the outputs from OrthoFinder to determine the number of overlapping (shared across species) transcripts across our four assemblies. Utilising the sister taxa in the present study provides a validation of the completeness of our *de novo* transcriptomes. In addition to assessing completeness of the final assemblies, we also applied OrthoFinder to assess and control completeness of our assemblies at each stage of the filtering pipeline, by comparing orthogroup size distribution within our salmon *de novo* assemblies relative to the Atlantic salmon RefSeq protein set (GCA_000233375.4).

Third, we quantified the extent of overlap between our four assemblies and the recently published Atlantic salmon reference genome [12]. Transcripts from our four assemblies were aligned to the Atlantic salmon reference genome ICSASG_v2 (GenBank: GCA_000233375.4 [54]) with GMAP (2016–11-07) [55], additionally using the ‘--cross-species’ parameter for heterospecific mapping.

Fourth, we compared completeness and similarity of the current assemblies for Arctic charr, brown trout and European whitefish to previously published transcriptomes for these three species. We compared our Arctic charr assembly to the Magnanou et al. [34] assembly of 34,690 transcripts (http://ngspipelines.toulouse.inra.fr:9021/, accession: E-MTAB-3522), and our brown trout and European whitefish assemblies to the corresponding species from the PhyloFish database [35] (75,338 and 74,701 transcripts respectively; http://phylofish.sigenae.org/index.html). First, we assessed how well full-length transcripts were represented in our assemblies compared to the previous transcriptomes. Full-length transcript reconstruction in the previous assemblies for each of the three species was evaluated following the same protocol described above for the four assemblies we present here. Separate BLASTP searches were made against the Atlantic salmon protein database for each species, and coverage was analysed using the ‘analyze_blastPlus_topHit_coverage.pl’ script. Second, we identified the transcript set overlap of the current compared to previously published conspecifics assemblies. To focus on protein coding sequences, we used TransDecoder to identify putative protein coding regions in the published assemblies [34, 35]. We then created BLAST databases from the predicted protein sequences for each species individually using NCBI-blast 2.2.30+ and performed BLASTP searches (−max_target_seqs 1, −evalue 1e-5) of our assemblies against those databases. Transcripts were considered ‘shared’ between the current and previous assemblies where our transcripts had alignment scores greater than 90% identity and 80% coverage.

### Annotation and gene ontology analysis

To provide comprehensive annotation of the final transcript sets, we compared our *de novo* assemblies against two annotation resources; the NBCI Atlantic salmon protein database and the SwissProt/UniProtKB [56] database. As described above, all four assemblies were BLASTP searched against the NCBI protein sequences for Atlantic salmon. Additional annotation was provided by aligning transcripts against the SwissProt database curated proteins using BLASTP (−max_target_seqs 1, −evalue 1e-3). SwissProt database alignments, and their corresponding UniProtKB accessions, were used to assign gene ontology (GO) functional annotation. All GO analyses were performed using the PANTHER (protein annotation through evolutionary relationship) classification tool [57].

We also performed a separate GO analysis on the subsets of transcripts that were identified by OrthoFinder as being ‘assembly-specific’ (i.e. only found in one species). Again GO annotation of the ‘assembly-specific’ transcripts was conducted with PANTHER, per the pipelines described above. GO analyses were used to assess whether the representation of functional categories differed between the subsets of ‘assembly-specific’ transcripts.

### Identification of paralogous sequences

We used two approaches to identify paralogous sequences in our salmonid assemblies. First, using the BUSCO tool, we determined the proportion of transcripts within each assembly that were likely paralogs, i.e. duplicated single-copy orthologs. Second, we used OrthoFinder algorithms that normalise all-v-all BLASTS for transcript length. This allows greater accuracy and recall of orthogroups compared to previous methods and therefore more precise detection of both orthologous and paralogous sequences [53].

## Results and discussion

### *de novo* transcriptome data and assembly

In this study, we present new, high-quality, protein-coding transcriptomes for four salmonid species: Atlantic salmon, brown trout, Arctic charr and European whitefish. RNA-seq libraries, generated from whole organism samples, yielded between 18 and 32 million paired-end reads per individual for eight individuals per species. Quality filtering (quality score > 30) removed approximately 11% of the raw reads. This resulted in high quality RNA-seq datasets, which contained between 180 and 210 M paired-end reads for each of the four species (Table 1).

**Table 1:** Summary of sequencing data used to generate the *de novo* transcriptome assemblies for each species based on paired-end (2 × 75 bp) Illumina sequencing

Feature	Atlantic salmon	Brown trout	Arctic charr	European whitefish
Total number of paired-end reads (~Million)	192	190	180	210
Average number of paired reads per sample (~Million)	23	24	23	26

### Quality assessment and filtering of assemblies

The initial *de novo* assemblies generated from Trinity ranged between 200,760 and 242,899 transcripts greater than 297 bp in length for the four species (Table 2). As a preliminary assessment of assembly quality, prior to filtering, we mapped the RNA-seq input reads for each species back to their transcriptome. In excess of 80% read mapping is considered to be indicative of a good quality assembly [45]. Respectively, for the Atlantic salmon, brown trout, Arctic charr and European whitefish assemblies we found that 89, 87, 90 and 91% of the reads successfully aligned.

**Table 2:** Assembly statistics for the Atlantic salmon, brown trout, Arctic charr and European whitefish *de novo* transcriptome assemblies

Feature	Atlantic salmon	Brown trout	Arctic charr	European whitefish
Number of base pairs in cleaned reads	64,909,254,125	67,282,460,986	65,841,176,651	73,342,359,278
Number of paired-end reads	191,977,874	190,239,319	180,232,708	209,578,198
Number of base pairs in initial assembly	182,476,550	179,378,175	156,753,048	162,053,186
Number of transcripts in initial assembly	235,515	242,899	200,760	209,920
Number of base pairs in final assembly	73,403,213	69,587,826	64,848,138	63,007,687
Number of transcripts in final assembly	36,505	35,736	33,126	33,697
Average transcript length (bp)	2011	1947	1957	1902
Minimum transcript length (bp)	297	297	297	298
Maximum transcript length (bp)	17,114	15,967	15,742	15,887
N50	2464	2393	2411	2325
N90	1115	1080	1087	1062

#### Removal of redundant transcripts

Redundant transcripts were identified using TransDecoder’s ORF predictions. After all predicted protein coding sequences were extracted they were filtered to select the ‘single-best’ ORF for each transcript, which reduced the number of assembled transcripts by about four-fold for each species, resulting in sets of 60,856 transcripts for Atlantic salmon, 60,943 for brown trout, 55,674 for Arctic charr and 57,734 for European whitefish. We clustered the remaining sequences with CD-Hit (100% amino acid identity), which collapsed around 12% of the transcripts. The resulting non-redundant assemblies consisted of 53,547 transcripts for Atlantic salmon, 53,804 for brown trout, 50,166 for Arctic charr and 50,994 for European whitefish. These results are consistent with the number of transcripts reported for previously published transcriptome assemblies for salmonids generated by independent research groups; lake whitefish (77,797 transcripts) [32], coho salmon (43,228 transcripts) [33], and Arctic charr (34,690 transcripts) [34] transcriptomes. However, in those other published assemblies, no annotation was found for around half of the transcripts. Therefore, we performed additional filtering and analyses on the *de novo* assemblies to produce comprehensive reference gene sets for each of the four species.

#### Reconstruction of full-length transcripts

**Fig. 3:**
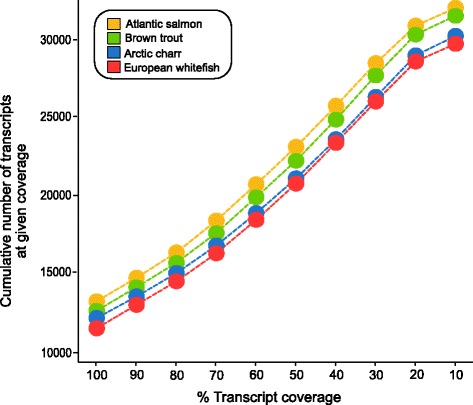
Cumulative number of genes with alignment to the NCBI protein database for Atlantic salmon (GCF_000233375.1) at a given coverage: Atlantic salmon (yellow), brown trout (green), Arctic charr (blue) and European whitefish (red)

A common problem in assembly of RNA-seq data is the high proportion of transcripts that are highly fragmented, due primarily to difficulties in determining accurate transcript boundaries [58, 59]. To produce assemblies that were representative of comprehensive gene sets in the current dataset, we examined the number of transcripts that were reconstructed to full length (100% alignment) or near full-length (> 70% alignment) by alignment to NCBI protein sequences for Atlantic salmon. For all four assemblies, 33–37% (between 11,099 and 13,546) of transcripts demonstrated complete (100%) alignment over 100% of their length. Furthermore, ~60% of the query transcripts aligned significantly (−evalue 1e-3) to the Atlantic salmon reference sequences over more than 70% of their length (Fig. 3). We detected no evidence of mapping bias, as might have been expected considering the varying level phylogenetic divergence (same species, same genus, different genera, different subfamilies) of our focal species from Atlantic salmon. Rather, we found that the number of reads mapped to the salmon reference was highly comparable across all four species (Fig. 3). This full-length transcript analysis was used to filter out and exclude fragmented transcripts (< 30% coverage).

After this filtering, the final assemblies contained 36,505 protein-coding transcripts for Atlantic salmon, 35,736 for brown trout, 33,126 for Arctic charr and 33,697 for European whitefish, which can be accessed through the NCBI transcriptome shotgun assembly database (TSA, https://www.ncbi.nlm.nih.gov/genbank/tsa/) (Additional file 1: Table S1). Summary statistics for each species assembly are shown in Table 2. N50 statistics were consistently high across all four assemblies (between 2325 and 2464). These results are comparable to, and in most cases outperform, those obtained for previously published *de novo* transcriptomes for salmonids [31–35]. Furthermore, the number of protein-coding sequences obtained in the final assemblies is consistent with the 37,206 annotated protein-coding genes that were estimated based on the recently published reference genome for Atlantic salmon [12].

### Assembly completeness and validation

While sized based metrics can be used to assess assembly continuity, they cannot be used to determine assembly completeness. We employed three robust, reference-based methods to evaluate and compare the completeness of the gene set of our four transcriptomes.

#### BUSCO analysis

First, protein gene set completeness was assessed using the BUSCO pipeline, which revealed that the majority of the Actinopterygian core genes had been successfully recovered in all four assemblies. Specifically, of the 4584 single-copy orthologs searched, we recovered 76% to 79% completely and 10 to 11% partially (Table 3). Only between 10 and 13% of the 4584 single-copy orthologs were classified as missing from our assemblies, indicating good coverage and high quality of the assembly of the protein-coding transcriptomes for these species. We found that BUSCO recovery in the current assemblies was three times greater than that identified for the PhyloFish assembly of the corresponding species. For both the brown trout and European whitefish assemblies presented here, we recovered 78 and 76% of the BUSCOs completely, whereas only 26% of BUSCOs were completely recovered in either of the previous trout and whitefish assemblies (Table 3). As expected, given the high-quality of the dataset, recovery for both ‘complete’ and ‘complete-duplicated’ BUSCOs was considerably higher for Atlantic salmon reference genome proteins, compared to either the current or previous assemblies, with 97% of BUSCOs completely recovered, of which 67% were duplicated (Table 3).

**Table 3:** Summary of the complete, duplicated, fragmented and missing orthologs inferred from Benchmarking Universal Single-Copy Orthologs (BUSCO) search against the 4584 single-copy orthologs for Actinopterygii

BUSCO statistic	Atlantic salmon	Brown trout	Arctic charr	European whitefish	PhyloFish Brown trout	PhyloFish European whitefish	NCBI Atlantic salmon RefSeq Proteins
Complete BUSCOs	3461 (79%)	3596 (78%)	3589 (78%)	3512 (76%)	1181 (26%)	1189 (26%)	4476 (97%)
Complete - single-copy BUSCOs	1900 (42%)	1897 (41%)	1988 (44%)	1938 (42%)	974 (21%)	995 (22%)	1398 (30%)
Complete – duplicated BUSCOs	1741 (37%)	1699 (37%)	1601 (34%)	1574 (34%)	207 (5%)	194 (4%)	3078 (67%)
Fragmented BUSCOs	439 (10%)	424 (10%)	431 (10%)	452 (11%)	155 (3%)	136 (3%)	80 (1.7%)
Missing BUSCOs	504 (10%)	564 (12%)	564 (12%)	620 (13%)	3248 (71%)	3259 (71%)	28 (0.6%)

However, we found that BUSCO recovery reported for the assemblies generated here was comparable to recent transcriptome studies using *de novo* methods, where recovery ranged between 68 and 95% [44, 60, 61]. BUSCO recovery tends to be highest when full organism and/or multiple developmental stages (e.g. 95% in ref. [44], 79 to 95% in ref. [60]) were used to generate the assemblies, as in the current study (Table 3), compared to those assembled from a select number of tissues (e.g. 68% in ref. [61]). Future combination of the current assemblies with RNA-seq data generated from different developmental stages could offer a promising means of producing transcriptomes with even greater levels of completion for these species. As far as we are aware, our study is the first to generate assemblies for these four species from entire specimens of juvenile fish and will therefore complement the published resources for these species, which have been generated from tissues of more mature fish [33–35].

#### OrthoFinder analysis

**Fig. 4:**
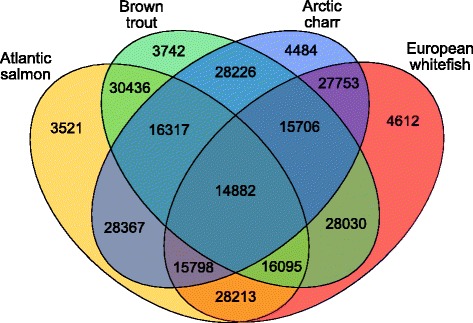
Venn diagram showing the number of overlapping orthologous protein groups between the four salmonid transcriptome assemblies. Orthologous proteins were identified with OrthoFinder

We used the program OrthoFinder as a second approach to evaluate assembly completeness based on sequence similarity. Orthogroup detection demonstrated considerable overlap in transcripts sequences across all four assemblies. Over 40% (14,882) of the transcripts that were identified as putative orthologs were shared across all four species. We also found that approximately 50% of the inferred orthogroups were represented by at least three species, and that over 84% of the orthologous transcripts identified in our four assemblies were shared by at least one of the other species’ assemblies (Fig. 4). As a result, a relatively low proportion of transcripts were identified as being unique to a given assembly, i.e. ‘assembly-specific’. We found that 3521 (10%) in Atlantic salmon, 3742 (10%) in brown trout, 4484 (14%) in Arctic charr and 4612 (14%) in European whitefish of all transcripts were found only in those species (Fig. 4). Additionally, we found that ~92% of the total transcripts in each of four assemblies were orthologous with at least one transcript from the Atlantic salmon RefSeq protein dataset (Additional file 2: Table S2). The marked level of sequence overlap observed between the four current transcriptomes, as well as between the published set of Atlantic salmon RefSeq proteins, further validates the completeness and quality of the assemblies presented here. This statement is further supported by the additional OrthoFinder analyses we performed comparing the orthogroup distribution size of the current salmon assembly (at all four filtering steps: unfiltered, after TransDecoder single-best ORF prediction, after CD-Hit clustering at 100% identity and after Trinity full-length transcript analysis (e.g. final version)) against the NCBI Atlantic salmon RefSeq proteins. Given the high quality of the recently published protein set for Atlantic salmon, we were able to empirically test whether we had successfully re-constructed a comprehensive set of orthologous transcripts in our assemblies. The results demonstrated good consistency, both between the present and existing protein sets for Atlantic salmon, as well as between subsequent filtering steps of the current salmon assembly (Additional file 3: Figure S2; Additional file 4: Table S3). Despite the relatively strict filtering we applied to the current assemblies, we found that only between 0.04 to 11% of the total orthogroups were lost during filtering. As such, these results further vindicate the quality of the assemblies we present here.

In addition to providing inference of assembly completeness, these results represent the first transcriptome-wide comparison of four ecologically and economically important salmonid species. Interestingly, we found no marked difference in the number of overlapping sequences between focal species with regards to their phylogenetic distance/proximity to each other (Figs. 1 and 4). The high number of putative orthologs observed in the current study is consistent with previous research investigating the molecular basis of phenotypic diversity in species rich cichlid fish complexes [62–64]. For example, transcriptomic diversity between ecologically divergent cichlid species, *Amphilophus astorquii* and *Amphilophus zaliosus*, using RNA-seq, found that over 50% of the 24,174 and 21,382 ESTs (respectively) were orthologous between the species [64]. These findings suggested limited or no genetic diversity at protein-coding regions among phenotypically diverse cichlid species. Here we present protein-coding regions across multiple species of salmonids and therefore can support new research into the molecular basis of phenotypic diversity in this group of highly diverse fishes.

#### Comparison against existing salmonid references

The final approach to assess assembly completeness was to assess alignment to relevant, independent references [50]. To do this, transcript sets for each of our four species were aligned to publically available resources for salmonids: the high-quality reference genome for Atlantic salmon and the recently published transcriptomes for Arctic charr [34], brown trout [35] and European whitefish [35].

##### Atlantic salmon reference genome

All four assemblies mapped to the salmon genome with high success of 98.5 to 99.5% (Table 4). There was no apparent relationship between mapping success and phylogenetic distance for brown trout (same genus as Atlantic salmon), Arctic charr (same subfamily but different genus from Atlantic salmon), or European whitefish (different subfamily from Atlantic salmon) (Table 4). However, our Atlantic salmon transcriptome predictably mapped with the highest success (99.5%) to the conspecific reference genome. This is consistent with the recent Atlantic salmon reference genome publication, which found that 98% of the NCBI mRNA sequences for Atlantic salmon aligned to the genome [12]. The comparable results demonstrated here indicated that we were able to successfully recover a set of high-confidence protein-coding genes in all four species’ transcriptomes.

**Table 4:** Alignment statistics of the new *de novo* transcriptomes mapping to the Atlantic salmon reference genome ICSASG_v2

Assembly	Number of transcripts in assembly	Total number of transcripts mapped	% Mapped transcripts
Atlantic salmon	36,505	36,305	99.5
Brown trout	35,736	35,186	98.5
Arctic charr	33,126	32,745	98.9
European whitefish	33,697	33,262	98.7

##### Previous transcriptome comparisons

First we assessed how successfully transcripts from the new and previous assemblies had been reconstructed to full (100% coverage) or near full (> 70% coverage) length compared to the NCBI protein database for Atlantic salmon. Of the seven assemblies, we found that the number of transcripts reconstructed to full-length was highest in the PhyloFish brown trout [35] assembly (19,404 transcripts), followed by the four current assemblies (11,099 to 13,546 transcripts), then the PhyloFish European whitefish [35] assembly (5073 transcripts), with the lowest number of full-length transcripts recovered in the Magnanou et al. Arctic charr [34] assembly (4411 transcripts) (Table 5, Additional file 5: Figure S1). However, with regard to the proportion of transcripts from the complete transcript sets for each assembly, we found that all four of our *de novo* assemblies had the greatest proportion of full and near full-length transcripts. Specifically, we achieved full-length (100%) reconstruction for 33 to 37% of transcripts and near to full-length recovery (> 70%) for 58 to 60% for the four assemblies generated here (Table 5). In contrast, we found that all three previous assemblies demonstrated a lower proportion recovery of full-length transcripts, with 12%, 26% and 7% recovery for the Arctic charr [34], brown trout [35] and European whitefish [35] assemblies, respectively. Brown trout had the highest absolute number of full transcripts (Table 5). Our analysis suggests that there are a higher proportion of fragmented transcripts in both the previous charr and trout assemblies, as well as higher levels of false duplicates and/or mis-assembled transcripts in all three previous assemblies. This further supports the quality of the new assemblies presented here and the relevance of their contribution to the currently available resources for salmonids.

**Table 5:** Comparison of full-length transcript reconstruction between the four current assemblies and three previously published transcriptomes for Arctic charr [34], brown trout [35] and European whitefish [35]. The table shows the number (percent) of transcripts from each assembly that aligned to the NCBI protein database for Atlantic salmon (GCF_000233375.1)

% Coverage against NCBI Atl. Salmon RefSeq Proteins	Atlantic salmon	Brown trout	Arctic charr	European whitefish	Magnanou et al. Arctic charr	PhyloFish Brown trout	PhyloFish European whitefish
100	13,546 (37%)	12,688 (36%)	12,127 (37%)	11,099 (33%)	4411 (12%)	19,624 (26%)	5073 (7%)
90–99	3072 (8%)	3232 (9%)	3220 (10%)	3659 (11%)	962 (3%)	4574 (6%)	1307 (2%)
80–89	2279 (6%)	2336 (7%)	2102 (6%)	2326 (7%)	777 (2%)	2306 (3%)	582 (1%)
70–79	2472 (7%)	2439 (7%)	2207 (7%)	2306 (7%)	933 (3%)	2185 (3%)	496 (1%)
60–69	3026 (8%)	2862 (8%)	2587 (8%)	2664 (8%)	1142 (3%)	2450 (3%)	508 (1%)
50–59	3458 (9%)	3461 (10%)	2966 (9%)	3172 (9%)	1514 (4%)	2883 (4%)	586 (1%)
40–49	3936 (10%)	3944 (11%)	3565 (11%)	3874 (11%)	2097 (6%)	3644 (5%)	738 (1%)
30–39	4716 (10%)	4774 (13%)	4352 (13%)	4597 (14%)	2587 (7%)	4348 (6%)	900 (1%)
20–29	0 (0%)	0 (0%)	0 (0%)	0 (0%)	3211 (9%)	4868 (6%)	1017 (1%)
10–19	0 (0%)	0 (0%)	0 (0%)	0 (0%)	3229 (9%)	4752 (6%)	908 (1%)
0–9	0 (0%)	0 (0%)	0 (0%)	0 (0%)	1709 (5%)	2932 (4%)	567 (1%)
No hit	0 (0%)	0 (0%)	0 (0%)	0 (0%)	12,118 (35%)	20,782 (28%)	62,019 (83%)

**Fig. 5:**
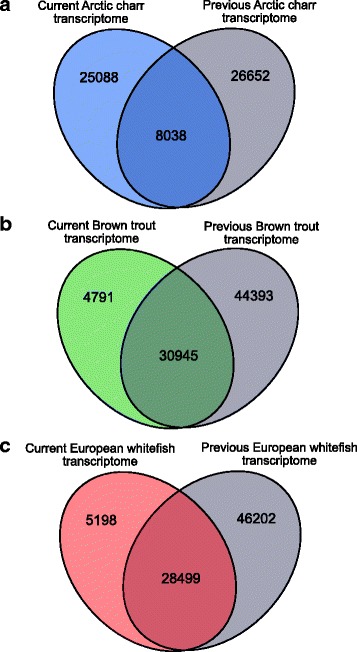
Venn diagrams showing the number of overlapping sequences between the current and previously published transcriptome assemblies for **a** Arctic charr (current vs. ref. [34]), **b** brown trout (current vs. ref. [35]) and **c** European whitefish (current vs. ref. [35])

Secondly, we used BLAST tools to compare sequence similarity between the current and previous assemblies for Arctic charr (current vs. [34]), brown trout (current vs. [35]) and European whitefish (current vs. [35]). Unexpectedly, we observed little overlap in the assembled transcripts between the current and previous charr assemblies (Fig. 5a). A total of 8038 sequences were identified as overlapping between the charr transcriptomes, which is representative of around 24% of the current assembly and 23% of the Magnanou et al. [34] assembly. For brown trout and European whitefish, the level of sequence similarity between the current and previous assemblies was considerably higher (Fig. 5b, c). In brown trout, 30,945 transcripts overlapped, representative of ~86% of the total transcripts from the current assembly and ~41% of the total PhyloFish transcripts. Similarly, for European whitefish we found sequence overlap for 28,499 transcripts, representative of ~85% of the total transcripts from the current assembly and ~38% of the PhyloFish transcripts.

Here we made no direct assessment of the cause of the differences between the charr assemblies and additionally the high proportion of transcripts that were unique to all three previous assemblies, however we offer several possible explanations. First and foremost, the data for the four current and PhyloFish assemblies were generated by Illumina sequencing platforms, whereas Magnanou et al. [34] used a 454-sequencing platform, which has variable read lengths and higher error rates. Second, different *de novo* assembly methods were used to build the transcriptomes. In both the present and PhyloFish [35] studies a de Bruijn graph algorithm approach was employed (using Trinity and Oases/Velvet assemblers respectively) while Magnanou et al.’s [34] study assembled with MIRA, which employs overlap graph methods [65]. Third, here we applied several steps of strict filtering to our assemblies so as to retain only protein-coding sequences, to help reduce noise and improve efficiency of downstream applications, whereas all three previous assemblies [34, 35] contain both coding and non-coding transcripts. Fourth, RNA-seq methods represent the transcriptome state at the point in time at which tissues are collected for RNA extraction. Gene activation and expression fluctuates throughout an organism’s life cycle, therefore the variation between the current and previous assemblies could be explained by the differences in the tissues used to generate them. In the current study RNA was extracted from whole organism samples of juvenile fish (~5 months), whereas both Magnanou et al. [34] and Pasquier et al. [35] used multiple tissues from mature adults, and additionally, Pasquier et al. [35] included embryonic tissue in their assemblies. Therefore, the transcripts from the current assemblies that did not overlap with the previous assemblies (25,088 transcripts for charr, 4791 for trout and 5198 for whitefish) can be used to complement and build upon the existing transcriptomic references for these species. Further, we performed several analyses to ensure high quality and completeness of our final transcript sets. Therefore, the subsets of transcripts found only in the current assemblies, compared to the three previous assemblies, offer an important and robust contribution to the currently available resources for these species, as well as other salmonids.

### Annotation and GO analysis

To provide comprehensive annotation of these four new transcriptomes, we conducted sequence homology searches against two different annotation resources. Using BLAST tools, we first compared transcripts against the NCBI Atlantic salmon protein database, as this represents the most established set of reference proteins that are publically available for salmonids at present. For this reason, successful alignment to the salmon protein database was also used to determine which transcripts were retained or discarded from our assemblies during optimisation (as detailed in Methods). Given that successful alignment to the NCBI Atlantic salmon protein database was used as part of our filtering pipeline, 100% of the final set of transcripts for all four assemblies are annotated to the salmon database (Table 6). Transcripts were further characterised by performing BLASTP searches against the UniProtKB/SwissProt curated proteins. Significant alignment (e-value 1e-3) for 95 to 96% of transcripts was found across our four assemblies (Table 6). The consistently complete or near complete annotation obtained across both protein databases gives us high confidence in the accuracy of the assembled transcripts.

**Table 6:** Number (and %) of transcripts with significant BLAST alignments to the databases listed

Database	Atlantic salmon	Brown trout	Arctic charr	European whitefish
NCBI Atlantic salmon proteins	36,505 (100%)	35,736 (100%)	33,126 (100%)	33,697 (100%)
SwissProt	34,843 (95%)	34,027 (95%)	31,607 (95%)	32,193 (96%)

The annotation statistics obtained for the four assemblies we present here are higher than those reported for previously published salmonid transcriptomes. Respectively, in the published lake whitefish [32], coho salmon [33] and Arctic charr [34] transcriptomes, 54, 40 and 48% of the transcripts were unannotated. Higher annotation success was obtained for the six salmonid species included in the PhyloFish database, with unannotated transcripts comprising just 9 to 15% of the assemblies [35]. Specifically, with regards to annotation against the well curated SwissProt database, we yielded significantly greater annotation (95 to 96%) compared to those obtained for the previous assemblies of our focal species; 3, 5.5 and 5.5% for Arctic charr [34], Brown trout [35] and European whitefish [35], respectively. The observed difference in annotation success between the current and previous assemblies is most likely due to the fact that, unlike previous studies, here we specifically filtered the assemblies to retain only protein-coding transcripts, with the aim of generating robust molecular resources to improve efficiency and accuracy of downstream genetic analyses. SwissProt/UniProtKB accessions are one of the most widely recognised by GO analysis softwares, therefore the high level of annotation against the SwissProt database makes our four assemblies very useful for future comparative analyses and downstream applications.

**Fig. 6:**
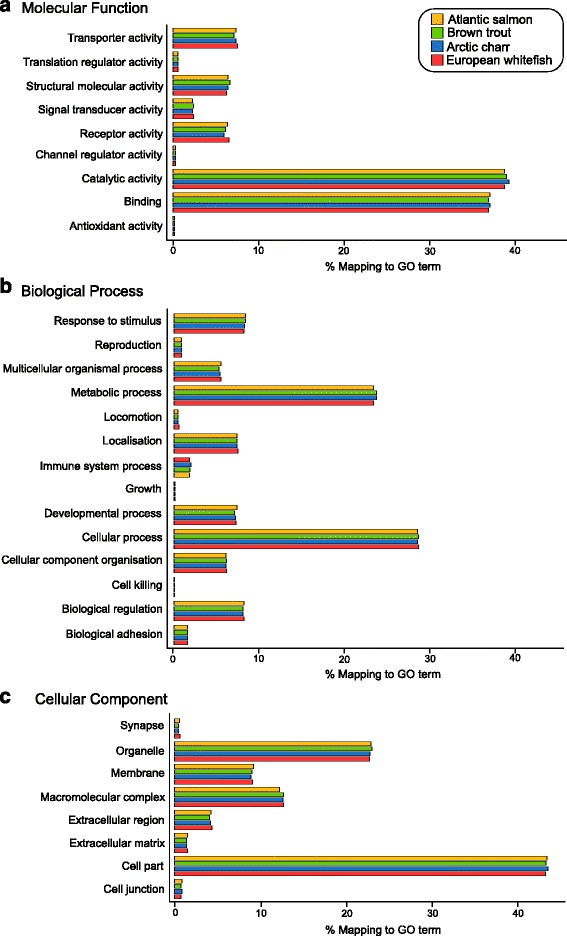
Proportions of gene ontology annotations for transcripts of Atlantic salmon (yellow), brown trout (green), Arctic charr (blue) and European whitefish (red): **a** molecular function, **b** biological process and **c** cellular component

Transcripts were functionally annotated based on their assigned UniProtKB/SwissProt gene symbols. We identified a wide range of GO terms in each assembly, indicating that molecular functions, biological processes and cellular components were well represented (Fig. 6 and Additional file 6: Table S4). High uniformity in GO profiles was observed across the four transcriptomes. These findings agree with previous research that reported high consistency of GO terms across multiple species, as well as across multiple phyla [62, 64, 66–68]. Consistency across the assemblies further indicates accuracy of the assemblies and the assigned annotations.

We performed a separate GO analysis on the ‘assembly-specific’ transcripts and observed no difference in the number and assortment of the gene ontology terms compared to the complete dataset (Fig. 6, Additional file 7: Figure S3). Further, the distribution of GOs for ‘assembly-specific’ transcript subsets was comparable to those obtained for the previous coho salmon [33] and Arctic charr [34] transcriptomes, and therefore further justifies their inclusion within the final assemblies.

Direct comparisons between the GOs assigned to the current transcriptomes and previously published transcriptomic data for salmonids is somewhat limited, given that the majority of transcriptomic research to date has focussed on subsets of GOs, related to specific biological and/or ecological questions [69–75]. Representation of GOs was consistent between the current and previous coho salmon [33] and Arctic charr [34] assemblies for all three categories (molecular function, biological processes and cellular component). The highest proportions of mapped GO terms for the current salmonid transcriptomes were related to binding (~37%) and catalytic activity (~39%) under “Molecular Function”, cellular (~29%) and metabolic (24%) processes under “Biological Process”, and cell part (~46%) and organelle (~24%) under “Cellular Component” (Fig. 6 and Additional file 6: Table S4). The consistency between the distribution of GOs in the new and previous transcriptomes suggests that we were able to successfully capture representative GO profiles for the four salmonids.

### Identification of paralogous sequences

Ancestral genome duplication increases the complexity of genetic studies in salmonids. Consequently, the need to distinguish between paralogous sequences and allelic variation presents a major challenge in generating comprehensive molecular resources for these species [10, 12, 33, 76]. Here we applied two methods to distinguish between orthologous and paralogous sequences in our transcriptome assemblies and by combining the results generate a robust approximation of the number of paralogous sequences. First, using the BUSCO tool, we found that 34 to 37% of the single-copy orthologs detected in our assemblies were duplicated (Table 3). Second, using OrthoFinder algorithms, we were able to estimate the total number of paralogous sequences present in our transcriptomic datasets by calculating the number of self-BLAST hits identified between transcripts within a single species (Table 7). Of the total number of transcripts in each of the species’ assemblies, we identified 37% in Atlantic salmon, 36% in brown trout, 34% in Arctic charr and 34% in European whitefish as putative paralogous transcripts. The high consistency in the proportion of paralogs detected by both methods applied here, BUSCO and OrthoFinder, increased our confidence that we were able to successfully identify ‘true’ paralogous sequences within our final assemblies. Further, to the best of our knowledge, the results presented here represent the most comprehensive identification of true paralogs within *de novo* assembled trancriptomes for salmonids, demonstrating a considerably higher capture rate than reported previously for the coho salmon transcriptome, where 29% of the assembled transcripts were identified as duplicates [33]. However it is important to note that although we have high confidence in our identified paralogs, they are not representative of the complete set of paralogs present across the genome.

**Table 7:** Number and percent of putative paralogous transcripts present in each species’ assembly, as identified by OrthoFinder algorithms

Assembly	Number of transcripts in assembly	Number of putative paralogous transcripts	% putative paralogous transcripts
Atlantic salmon	36,505	13,474	37
Brown trout	35,736	12,746	36
Arctic charr	33,126	11,381	34
European whitefish	33,697	11,518	34

Publication of the high-quality reference genome for Atlantic salmon has provided invaluable insight into the rediploidization process and the evolutionary fate of duplicated genes within the salmonid genome [12]. Lien et al. [12] found that 55% of the duplicated genes created during the salmonid-specific WGD event have been retained as two functional copies in the genome. This corresponds with a previous study investigating rediploidization in the rainbow trout genome, in which it was reported that 48% of duplicated genes had been retained [10]. The increased complexity of the salmonid genomes makes it difficult to distinguish between true paralogs and duplicated sequences that result from sequencing error and mis-assembly. The reduced proportion of duplicate genes (34 to 37%) identified in the current study is likely a result of the current limitations for *de novo* assembly algorithms. Specifically, *de novo* assemblers, such as Trinity, are not able to distinguish between similar paralogs, therefore reconstruction of the complete set of paralogs for species with such highly duplicated genomes remains a major challenge. Discerning between true and false ‘duplicate’ sequences is biologically and analytically complex, and there is currently no standard pipeline for identifying paralogs within *de novo* assembled transcriptomes. Therefore, our aim in the present study was to balance the trade-off between the removal of redundant duplicate sequences and retaining as best as possible a representative set of ‘true’ paralogs. The incomplete set of paralogs captured in *de novo* assemblies presented here (34 to 37%, compared to over 48% in genome inferred data) further highlights the need to continually develop molecular resources for salmonids, and additionally illustrates how our transcriptomes can be used to complement the existing the resources for these species.

## Conclusions

This study presents the release of new protein-coding transcriptomes for four ecologically and economically important salmonids; Atlantic salmon, brown trout, Arctic charr and European whitefish. As such, this research represents an important contribution to the existing genomic resources for salmonid taxa. Furthermore, we provide a comprehensive overview and characterization of the generated transcriptomes, as well as presenting a comparison across these four species. The marked level of continuity and completeness of the transcriptomes is highly supported by several methods of quantitative and qualitative assessment. The thorough optimisation performed will facilitate more efficient and accurate future analyses and downstream applications on gene expression and sequence evolution. Therefore, the current transcriptomes provide robust resources for future genomic investigation in these species, and additionally provide valuable tools, which can be used to inform comparisons on other salmonid species of evolutionary, ecological and economic interest.

## Additional files

Additional file 1: Table S1. NCBI SRA and TSA accessions for raw read data and assemblies (respectively), for all four species. (PDF 58 kb)

Additional file 2: Table S2. Database of the orthogroups containing Atlantic salmon RefSeq proteins (GCF_000233375.1), and the corresponding transcripts from four *de novo* protein-coding transcriptomes presented here; for Atlantic salmon, Brown trout, Arctic charr and European whitefish. (TSV 8775 kb)

Additional file 3: Figure S2. Comparison of orthogroup size distribution between the current *de novo* assembly for Atlantic salmon, at each stage of filtering, relative to Atlantic salmon reference genome proteins (GCF_000233375.4). (PDF 57 kb)

Additional file 4: Table S3. Distribution of orthologs in the current Atlantic salmon *de novo* assembly compared to the distribution of orthologs in the NCBI Atlantic salmon RefSeq protein dataset (GCF_000233375.1). OrthoFinder results were filtered to retain only OrthoGroups with at least one RefSeq salmon protein present. (PDF 228 kb)

Additional file 5: Figure S1. Comparison of full-length transcript reconstruction between the four current assemblies and three previously published transcriptomes; Magnanou et al.’s Arctic charr assembly [34], and the PhyloFish DB brown trout and European whitefish assemblies [35]. Cumulative number of unique matching proteins that aligned to the NCBI protein database for Atlantic salmon (GCF_000233375.1) at a given coverage: Atlantic salmon (yellow), brown trout (green), Arctic charr (blue), European whitefish (red), Magnanou et al. Arctic charr transcriptome (black), PhyloFish brown trout (dark grey), and PhyloFish European whitefish (light grey). (PDF 35 kb)

Additional file 6: Table S4. Number and proportion of gene ontology annotations assigned to each species’ transcriptome. (PDF 246 kb)

Additional file 7: Figure S3. GO analysis performed on the subsets of ‘species-specific’ transcripts for each of the four assemblies. (PDF 34 kb)

## Abbreviations

BLAST: Basic local alignment search tool
bp: base pair
BUSCO: Benchmarking set of Universal Single-Copy Orthologs
GO: Gene ontology
NCBI: National Centre for Biotechnology Information
ORF: Open reading frame
RIN: RNA integrity number
WGD: whole genome duplication
