# Correction to: De novo transcriptome assembly, annotation and comparison of four ecological and evolutionary model salmonid fish species

**DOI:** 10.1186/s12864-018-4840-5

**Published:** 2018-06-11

**Authors:** Madeleine Carruthers, Andrey A. Yurchenko, Julian J. Augley, Colin E. Adams, Pawel Herzyk, Kathryn R. Elmer

**Affiliations:** 1 Institute of Biodiversity, Animal Health and Comparative Medicine, College of Medical, Veterinary and Life Sciences, University of Glasgow, G12 8QQ, Glasgow, UK; 2 Glasgow Polyomics, Wolfson Wohl Cancer Research Centre, University of Glasgow, G61 1QH, Glasgow, UK; 3 Scottish Centre for Ecology and the Natural Environment, University of Glasgow, Rowardennan, G63 0AW UK; 4 Present Address: Fios Genomics Ltd., Nine Edinburgh Bioquarter, 9 Little France Road, Edinburgh, EH16 4UX UK; 5 Institute of Molecular, Cell and Systems Biology, College of Medical, Veterinary and Life Sciences, University of Glasgow, G12 8QQ, Glasgow, UK

## Correction

Following the publication of this article [1], the authors found that they incorrectly reported the BUSCO completeness for the PhyloFish brown trout and European whitefish transcriptomes. This was due to an error in their TransDecoder pipeline and restricted to those two datasets and their interpretation. Carruthers et al. apologise for this misreported result and thank the authors of the PhyloFish database for bringing it to their attention.

The correct version of Table 3 has been included in this correction.

**Table 3:** Summary of the complete, duplicated, fragmented and missing orthologs inferred from Benchmarking Universal Single-Copy Orthologs (BUSCO) search against the 4584 single-copy orthologs for Actinopterygii

BUSCO statistic	Atlantic salmon	Brown trout	Arctic charr	European whitefish	PhyloFish brown trout	PhyloFish European whitefish	NCBI Atlantic salmon RefSeq Proteins
Complete BUSCOs	3461 (79%)	3596 (78%)	3589 (78%)	3512 (76%)	4254 (92%)	4202 (91%)	4476 (97%)
Complete -single-copy BUSCOs	1900 (42%)	1897 (41%)	1988 (44%)	1938 (42%)	2327 (50%)	2444 (53%)	1398 (30%)
Complete - duplicated BUSCOs	1741 (37%)	1699 (37%)	1601 (34%)	1574 (34%)	1927 (43%)	1758 (38%)	3078 (67%)
Fragmented BUSCOs	439 (10%)	424 (10%)	431 (10%)	452 (11%)	83 (1.8%)	113 (2.4%)	80 (1.7%)
Missing BUSCOs	504 (10%)	564 (12%)	564 (12%)	620 (13%)	247 (5.3%)	269 (5.8%)	28 (0.6%)
