# Supplementary material for: De novo transcriptome assembly, annotation and comparison of four ecological and evolutionary model salmonid fish species
Source: BMC Genomics. 2018 Jan 8;19:32. doi: 10.1186/s12864-017-4379-x (PMC5759245; doi:10.1186/s12864-017-4379-x)
Supplement: Supplementary file 1 — NCBI SRA and TSA accessions for raw read data and assemblies (respectively), for all four species. (PDF 58 kb) [file 12864_2017_4379_MOESM1_ESM.pdf]

Table S1 Number of paired-end reads used to generate transcriptomes, and the number of transcripts in the final assemblies, with their corresponding accession numbers in the NCBI SRA and TSA databases.

Species	Number of clean paired-end sequencing reads	SRA database accession numbers	Number of transcripts in assembly	TSA database accession number
Atlantic salmon	192 M	SRR6321799, SRR6321800, SRR6321801, SRR6321802, SRR6321805, SRR6321806, SRR6321807, SRR6321808	36,505	GGAQ01000000
Brown trout	190 M	SRR6321795, SRR6321796, SRR6321797, SRR6321798, SRR6321803, SRR6321804, SRR6321815, SRR6321816	35,736	GGAR01000000
Arctic charr	180 M	SRR6321809, SRR6321810, SRR6321811, SRR6321812, SRR6321813, SRR6321814, SRR6321825, SRR6321826	33,126	GGAP01000000
European whitefish	210 M	SRR6321817, SRR6321818, SRR6321819, SRR6321820, SRR6321821, SRR6321822, SRR6321823, SRR6321824	33,697	GGAO01000000
