# Supplementary material for: De novo transcriptome assembly, annotation and comparison of four ecological and evolutionary model salmonid fish species
Source: BMC Genomics. 2018 Jan 8;19:32. doi: 10.1186/s12864-017-4379-x (PMC5759245; doi:10.1186/s12864-017-4379-x)
Supplement: Supplementary file 3 — Comparison of orthogroup size distribution between the current de novo assembly for Atlantic salmon, at each stage of filtering, relative to Atlantic salmon reference genome proteins (GCF_000233375.4). (PDF 57 kb) [file 12864_2017_4379_MOESM3_ESM.pdf]

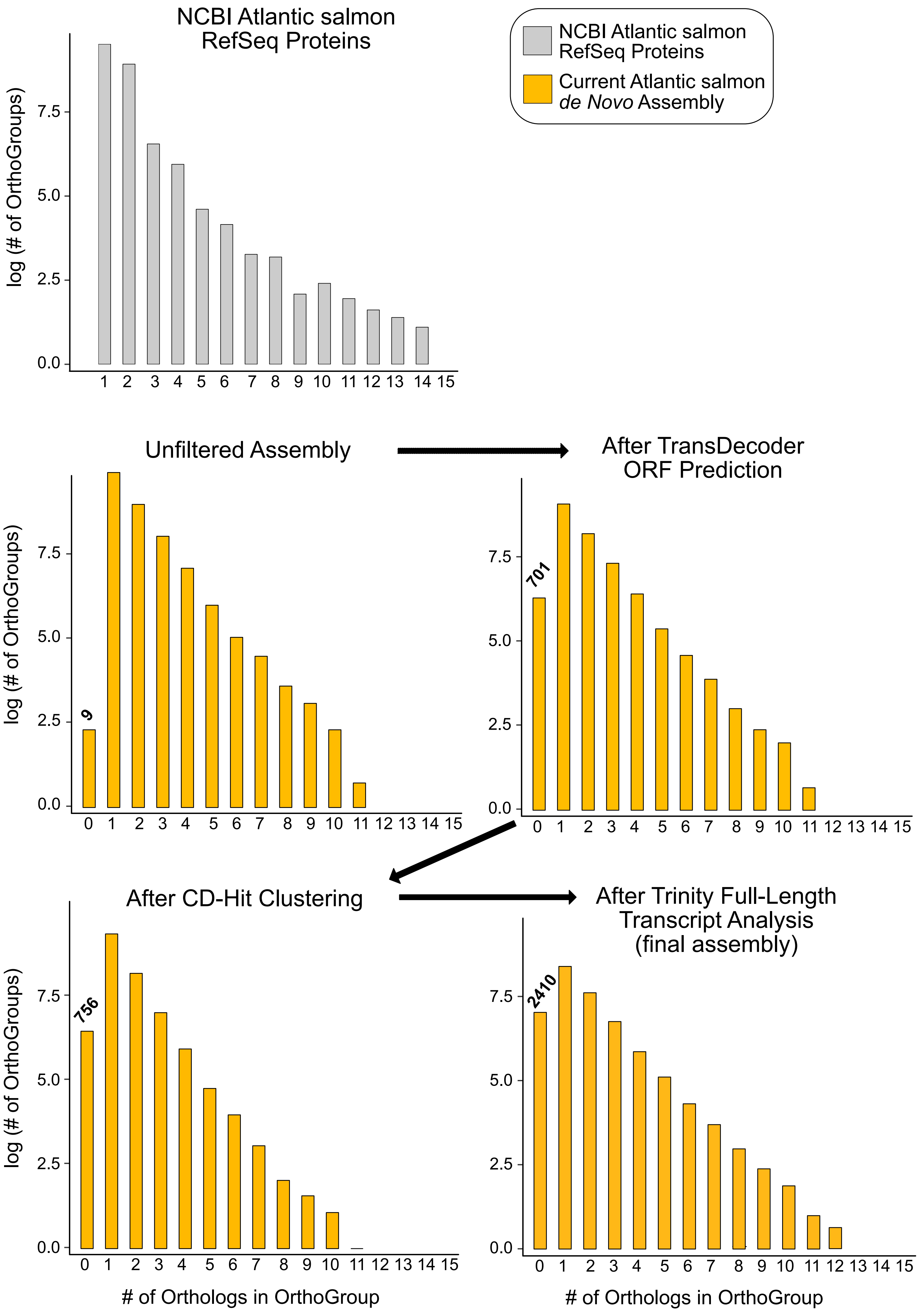
NCBI Atlantic salmon
RefSeq Proteins
NCBI Atlantic salmon
RefSeq Proteins
Current Atlantic salmon
de Novo Assembly
log (# of OrthoGroups)
Unfiltered Assembly
9
After TransDecoder
ORF Prediction
701
After CD-Hit Clustering
756
After Trinity Full-Length
Transcript Analysis
(final assembly)
2410
# of Orthologs in OrthoGroup
# of Orthologs in OrthoGroup
