# Supplementary material for: De novo transcriptome assembly, annotation and comparison of four ecological and evolutionary model salmonid fish species
Source: BMC Genomics. 2018 Jan 8;19:32. doi: 10.1186/s12864-017-4379-x (PMC5759245; doi:10.1186/s12864-017-4379-x)
Supplement: Supplementary file 4 — Distribution of orthologs in the current Atlantic salmon de novo assembly compared to the distribution of orthologs in the NCBI Atlantic salmon RefSeq protein dataset (GCF_000233375.1). OrthoFinder results were filtered to retain only OrthoGroups with at least one RefSeq salmon protein present. (PDF 228 kb) [file 12864_2017_4379_MOESM4_ESM.pdf]

Additional file 4: **Table S3** Distribution of orthologs in the current Atlantic salmon *de novo* assembly compared to the distribution of orthologs in the NCBI Atlantic salmon RefSeq protein dataset (GCF_000233375.1). OrthoFinder results were filtered to retain only OrthoGroups with at least one RefSeq salmon protein present.

Number of Orthologs in Orthogroup	Number of Orthogroups with given number of Orthologs in Salmon *de Novo* at Different Filtering Steps				NCBI Atlantic salmon RefSeq Proteins
	Unfiltered	After TransDecoder Single Best ORF Prediction	After CD-Hit Clstering at 100% Identity	After Trinity Full-Length Transcript Analysis (final assembly)	
0	9	701	756	2410	N/A
1	13,002	12,737	14,693	10934	13221
2	5,279	5,097	4,413	4595	7312
3	2,143	2,044	1,331	1782	686
4	868	793	440	661	375
5	305	270	132	287	99
6	123	119	59	119	63
7	72	57	23	60	26
8	31	23	8	27	24
9	19	12	5	14	8
10	9	8	3	8	11
11	2	2	1	3	7
12	0	0	0	2	5
13	0	0	0	1	4
14	0	0	0	0	3
15	0	1	0	1	0
16	0	0	0	0	1
17	0	0	0	2	0
18	0	0	0	0	0
19	0	0	0	0	2
20	0	0	0	0	3
21	1	0	0	0	1
22	0	0	0	0	1
23	0	0	0	0	0
24	0	0	0	0	1
25	0	0	0	0	0
26	0	0	0	0	3
27	0	0	0	0	1
28	0	0	0	0	0
29	1	0	0	0	1
30	0	0	0	0	0
