# Supplementary figures and images for: De novo transcriptome assembly, annotation and comparison of four ecological and evolutionary model salmonid fish species
Source: BMC Genomics. 2018 Jan 8;19:32. doi: 10.1186/s12864-017-4379-x (PMC5759245; doi:10.1186/s12864-017-4379-x)

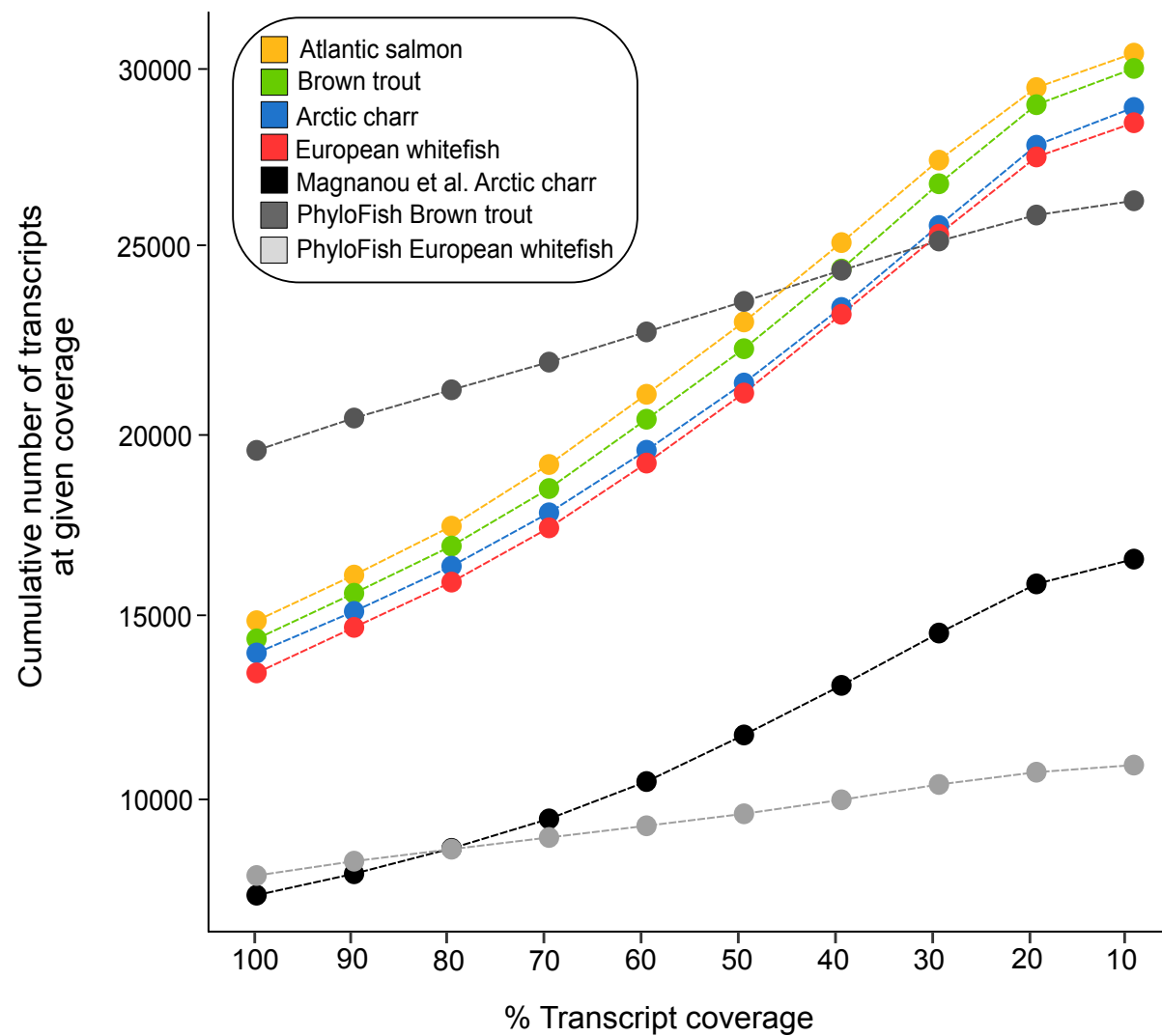

Supplement: Supplementary file 5 — Comparison of full-length transcript reconstruction between the four current assemblies and three previously published transcriptomes; Magnanou et al.’s Arctic charr assembly [34], and the PhyloFish DB brown trout and European whitefish assemblies [35]. Cumulative number of unique matching proteins that aligned to the NCBI protein database for Atlantic salmon (GCF_000233375.1) at a given coverage: Atlantic salmon (yellow), brown trout (green), Arctic charr (blue), European whitefish (red), Magnanou et al. Arctic charr transcriptome (black), PhyloFish brown trout (dark grey), and PhyloFish European whitefish (light grey). (PDF 35 kb) [file 12864_2017_4379_MOESM5_ESM.pdf]

## a) Molecular Function

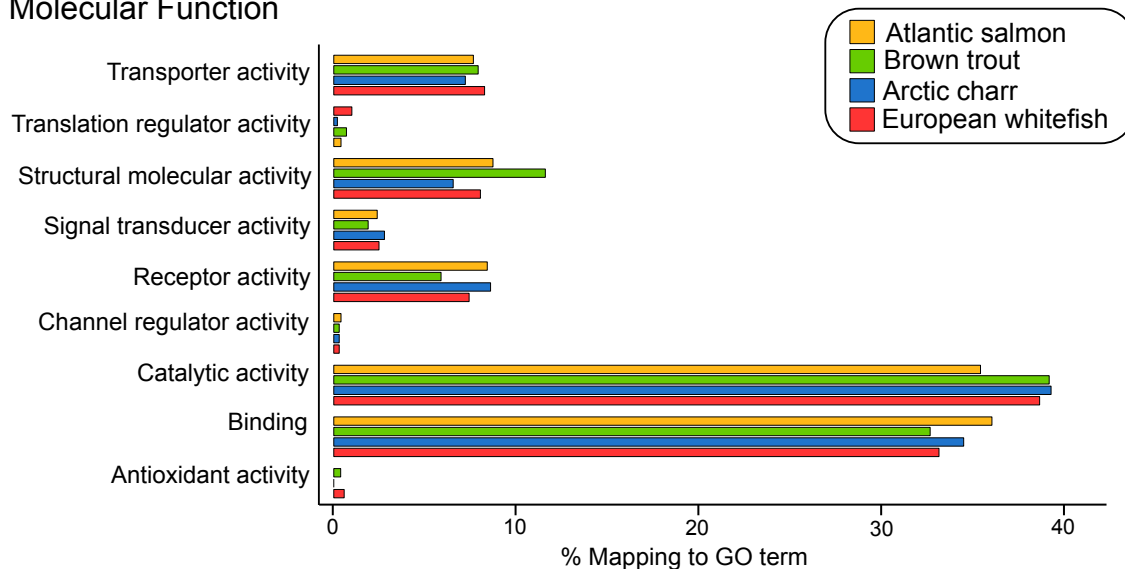

## b) Biological Process

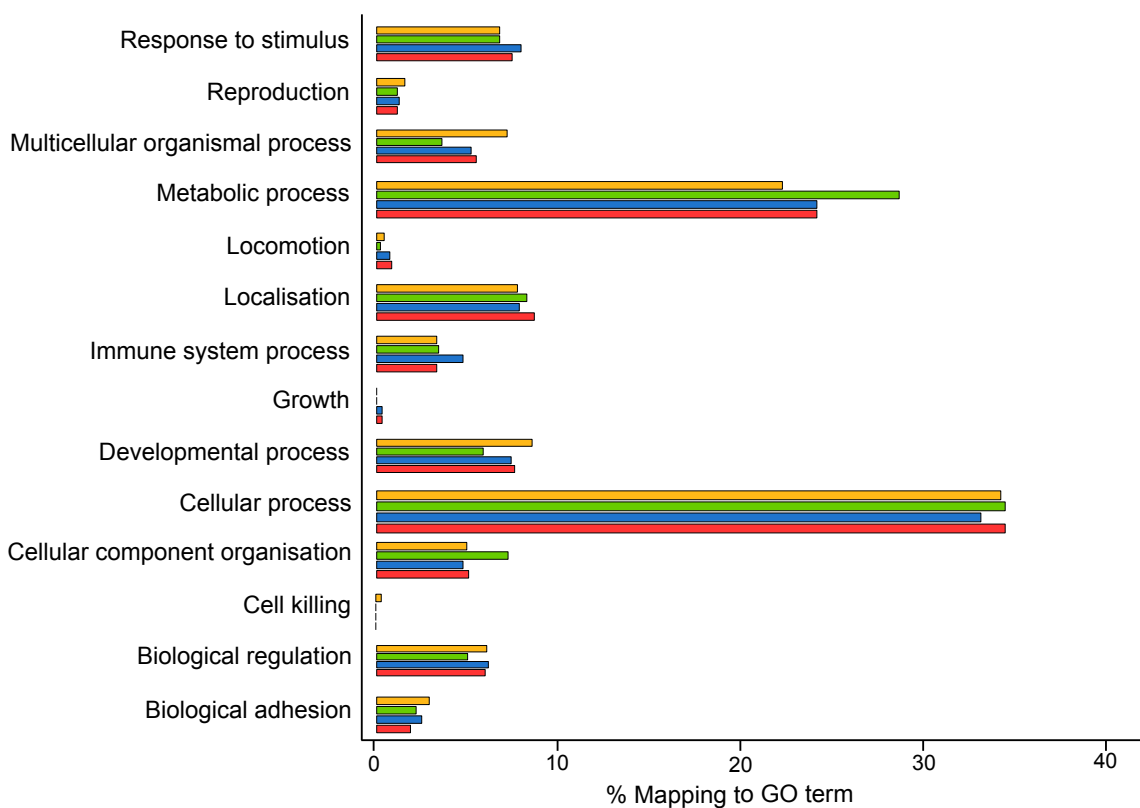

## c) Cellular Component

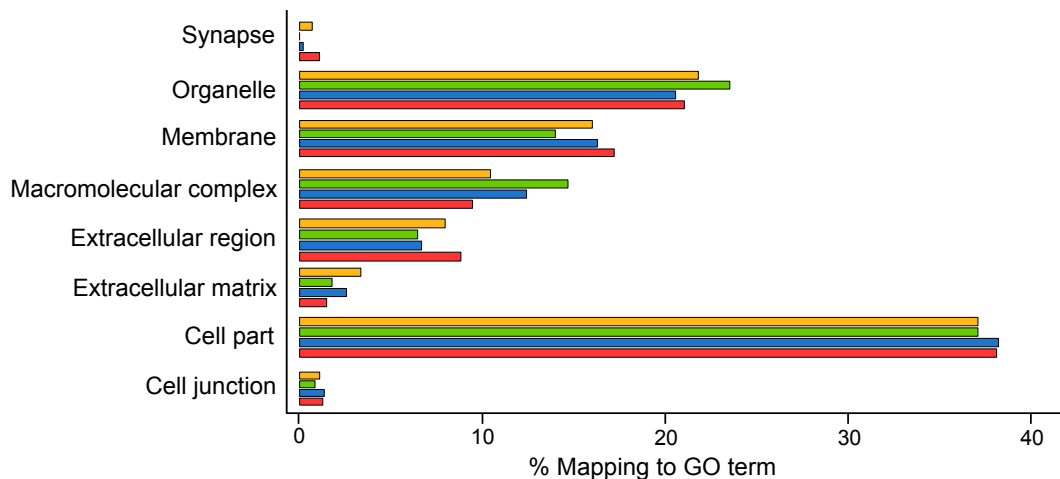

Supplement: Supplementary file 7 — GO analysis performed on the subsets of ‘species-specific’ transcripts for each of the four assemblies. (PDF 34 kb) [file 12864_2017_4379_MOESM7_ESM.pdf]
