# Supplementary material for: De novo transcriptome assembly, annotation and comparison of four ecological and evolutionary model salmonid fish species
Source: BMC Genomics. 2018 Jan 8;19:32. doi: 10.1186/s12864-017-4379-x (PMC5759245; doi:10.1186/s12864-017-4379-x)
Supplement: Supplementary file 6 — Number and proportion of gene ontology annotations assigned to each species’ transcriptome. (PDF 246 kb) [file 12864_2017_4379_MOESM6_ESM.pdf]

Table S4 GO annotation (number and proportion of GO terms) assigned to the de novo transcriptomes for each of the four species.

Species	Molecular Function	# of Genes Mapped to GO Term	% Genes Mapped to GO Term
Atlantic salmon	transporter activity	824	7.4
Atlantic salmon	translation regulator activity	66	0.6
Atlantic salmon	catalytic activity	4318	39.0
Atlantic salmon	channel regulator activity	30	0.3
Atlantic salmon	receptor activity	712	6.4
Atlantic salmon	signal transducer activity	257	2.3
Atlantic salmon	antioxidant activity	19	0.2
Atlantic salmon	structural molecule activity	720	6.5
Atlantic salmon	binding	4135	37.3
Brown trout	transporter activity	788	7.2
Brown trout	translation regulator activity	68	0.6
Brown trout	catalytic activity	4292	39.2
Brown trout	channel regulator activity	35	0.3
Brown trout	receptor activity	683	6.2
Brown trout	signal transducer activity	262	2.4
Brown trout	antioxidant activity	24	0.2
Brown trout	structural molecule activity	733	6.7
Brown trout	binding	4059	37.1
Arctic charr	transporter activity	782	7.4
Arctic charr	translation regulator activity	64	0.6
Arctic charr	catalytic activity	4199	39.5
Arctic charr	channel regulator activity	30	0.3
Arctic charr	receptor activity	641	6.0
Arctic charr	signal transducer activity	242	2.3
Arctic charr	antioxidant activity	24	0.2
Arctic charr	structural molecule activity	690	6.5
Arctic charr	binding	3966	37.3
European whitefish	transporter activity	820	7.6
European whitefish	translation regulator activity	61	0.6
European whitefish	catalytic activity	4225	39.0
European whitefish	channel regulator activity	30	0.3
European whitefish	receptor activity	709	6.6
European whitefish	signal transducer activity	257	2.4
European whitefish	antioxidant activity	22	0.2
European whitefish	structural molecule activity	681	6.3
European whitefish	binding	4016	37.1
**Species**	**Biological Process**	**Number of Genes Mapped to GO Term**	**% Genes Mapped to GO Term**
Atlantic salmon	cellular component organization or biogenesis	1392	6.1
Atlantic salmon	cellular process	6538	28.6

Atlantic salmon	localization	1700	7.4
Atlantic salmon	reproduction	202	0.9
Atlantic salmon	biological regulation	1863	8.2
Atlantic salmon	response to stimulus	1923	8.4
Atlantic salmon	developmental process	1698	7.4
Atlantic salmon	multicellular organismal process	1246	5.5
Atlantic salmon	locomotion	123	0.5
Atlantic salmon	biological adhesion	375	1.6
Atlantic salmon	metabolic process	5345	23.4
Atlantic salmon	growth	17	0.1
Atlantic salmon	immune system process	420	1.8
Atlantic salmon	cell killing	6	0.0
Brown trout	cellular component organization or biogenesis	1393	6.2
Brown trout	cellular process	6433	28.7
Brown trout	localization	1653	7.4
Brown trout	reproduction	208	0.9
Brown trout	biological regulation	1810	8.1
Brown trout	response to stimulus	1875	8.4
Brown trout	developmental process	1599	7.1
Brown trout	multicellular organismal process	1196	5.3
Brown trout	locomotion	111	0.5
Brown trout	biological adhesion	364	1.6
Brown trout	metabolic process	5347	23.8
Brown trout	growth	19	0.1
Brown trout	immune system process	423	1.9
Brown trout	cell killing	6	0.0
Arctic charr	cellular component organization or biogenesis	1335	6.1
Arctic charr	cellular process	6258	28.6
Arctic charr	localization	1612	7.4
Arctic charr	reproduction	206	0.9
Arctic charr	biological regulation	1765	8.1
Arctic charr	response to stimulus	1816	8.3
Arctic charr	developmental process	1573	7.2
Arctic charr	multicellular organismal process	1188	5.4
Arctic charr	locomotion	110	0.5
Arctic charr	biological adhesion	358	1.6
Arctic charr	metabolic process	5202	23.8
Arctic charr	growth	14	0.1
Arctic charr	immune system process	427	2.0
Arctic charr	cell killing	8	0.0
European whitefish	cellular component organization or biogenesis	1386	6.2
European whitefish	cellular process	6385	28.7
European whitefish	localization	1668	7.5
European whitefish	reproduction	197	0.9
European whitefish	biological regulation	1834	8.2
European whitefish	response to stimulus	1836	8.2

European whitefish	developmental process	1616	7.3
European whitefish	multicellular organismal process	1216	5.5
European whitefish	locomotion	126	0.6
European whitefish	biological adhesion	358	1.6
European whitefish	metabolic process	5224	23.4
European whitefish	growth	13	0.1
European whitefish	immune system process	411	1.8
European whitefish	cell killing	4	0.0

Species	Cellular Component	Number of Genes Mapped to GO Term	% Genes Mapped to GO Term
Atlantic salmon	synapse	60	0.6
Atlantic salmon	cell junction	88	0.9
Atlantic salmon	membrane	959	9.7
Atlantic salmon	macromolecular complex	1276	12.9
Atlantic salmon	extracellular matrix	156	1.6
Atlantic salmon	cell part	4513	45.7
Atlantic salmon	organelle	2376	24.1
Atlantic salmon	extracellular region	449	4.5
Brown trout	synapse	54	0.5
Brown trout	cell junction	84	0.8
Brown trout	membrane	944	9.5
Brown trout	macromolecular complex	1324	13.4
Brown trout	extracellular matrix	149	1.5
Brown trout	cell part	4517	45.6
Brown trout	organelle	2399	24.2
Brown trout	extracellular region	430	4.3
Arctic charr	synapse	51	0.5
Arctic charr	cell junction	89	0.9
Arctic charr	membrane	891	9.4
Arctic charr	macromolecular complex	1262	13.3
Arctic charr	extracellular matrix	146	1.5
Arctic charr	cell part	4337	45.8
Arctic charr	organelle	2267	24.0
Arctic charr	extracellular region	418	4.4
European whitefish	synapse	67	0.7
European whitefish	cell junction	79	0.8
European whitefish	membrane	928	9.6
European whitefish	macromolecular complex	1295	13.4
European whitefish	extracellular matrix	156	1.6
European whitefish	cell part	4403	45.5
European whitefish	organelle	2309	23.9
European whitefish	extracellular region	441	4.6
